# Diagnostic Accuracy and Histopathological Correlation of Suspected Barrett’s Esophagus in Patients Undergoing Endoscopy for Dyspeptic Symptoms: A Retrospective Cross-Sectional Study

**DOI:** 10.34172/aim.34695

**Published:** 2025-11-01

**Authors:** Yavuz Emre Parlar, Zahide Şimşek

**Affiliations:** ^1^Department of Gastroenterology, Sincan Training and Research Hospital, Ankara, Turkey; ^2^Department of Gastroenterology, Bayindir Hospital, Ankara, Turkey

**Keywords:** Barret’s esophagus, Dyspepsia, Endoscopy, Gastroesophageal reflux

## Abstract

**Background:**

Barrett’s esophagus (BE) is a premalignant condition resulting from chronic gastroesophageal reflux disease (GERD), associated with increased risk of esophageal adenocarcinoma. Endoscopic identification of BE remains imperfect without histological confirmation. This study evaluates the diagnostic accuracy of endoscopy in detecting BE and identifies its associated factors using data from a large cohort of dyspeptic patients.

**Methods:**

In this retrospective study, we reviewed 41,268 adults undergoing upper gastrointestinal endoscopy for dyspeptic symptoms between 2014 and 2018. Among them, 840 had suspected BE. Biopsies were obtained per the Seattle protocol and assessed for intestinal metaplasia by expert pathologists. Clinical and endoscopic features and *Helicobacter pylori* status were analyzed.

**Results:**

Histological confirmation was achieved in 423 of 840 patients (50.4%). Short-segment BE comprised 97.4% of cases. Multivariate analysis identified age≥50 years, male sex, presence of hiatal hernia, and absence of *H. pylori* as independent predictors of histologically confirmed BE (*P*<0.05 for all). *H. pylori* prevalence was significantly lower in BE patients (26.2%) compared to non-BE patients (47.7%).

**Conclusion:**

Endoscopic suspicion alone significantly overestimates BE prevalence, particularly for short-segment BE. Systematic biopsies remain essential for accurate diagnosis. The inverse association between *H. pylori* and BE suggests that *H. pylori* infection may be linked with a lower prevalence of BE. Targeted screening strategies are needed, especially in high-risk populations.

## Introduction

 Barrett’s esophagus (BE) represents a metaplastic change from squamous to columnar epithelium with intestinal metaplasia in the distal esophagus, primarily driven by chronic gastroesophageal reflux disease (GERD).^1^ Its frequency is 1.5% in the general population, and 15% in patients with GERD.^2^ BE is classified as long-segment (columnar mucosa length seen in the esophagus > 3 cm) and short-segment (columnar mucosa length seen in the esophagus < 3 cm). It is a well-established precursor to esophageal adenocarcinoma, a malignancy with poor prognosis and rising incidence in many regions including Turkey. Accurate detection and monitoring of BE are essential to reduce the risk of progression to dysplasia or carcinoma.^3^

 The diagnosis of BE requires both endoscopic identification of columnar-lined esophageal mucosa extending at least 1 cm above the gastroesophageal junction and histological confirmation of intestinal metaplasia in biopsies from the affected area. Although endoscopy remains the cornerstone of BE diagnosis, distinguishing columnar epithelium due to BE from gastric cardia or inflammatory changes can be difficult, especially in short-segment BE. Histological confirmation through systematic biopsies remains the gold standard.^4^ In this study, we present one of the largest retrospective patient series from Turkey evaluated for BE to determine the diagnostic concordance between endoscopic suspicion and histopathological confirmation. We also examine its associated demographic and clinical features, with special focus on the controversial relationship between BE and *Helicobacter pylori* infection.

## Materials and Methods

###  Study Design

 In this study, we retrospectively reviewed the medical records of 41,268 adult patients ( > 18 years) who underwent upper gastrointestinal endoscopy for dyspeptic complaints between January 2014 and April 2018 at the Gastroenterology Department of Ankara Dışkapı Yıldırım Beyazıt Training and Research Hospital. Of these, 840 were reported as having endoscopic findings suggestive of BE, based on visualization of columnar-lined mucosa extending ≥ 1 cm above the gastroesophageal junction. Demographic characteristics, dyspeptic symptoms, smoking and alcohol consumption, proton pump inhibitor (PPI) use, endoscopic findings (including esophagitis, hiatal hernia (HH), peptic ulcer, gastritis, and lower esophageal sphincter (LES) laxity, as well as histopathological results were recorded for all patients. Patients with gastric carcinoma, gastrointestinal bleeding, and coagulopathy were excluded from the study. The study protocol was approved by the hospital’s local ethics committee (Approval Date: July 16, 2018; Protocol no: B-18993).

###  Endoscopic Evaluation

 Endoscopic examinations were performed by gastroenterologists using a standard endoscope (Fujinon EPX-4400, System WR, Fujifilm, Japan). During endoscopy, the esophagogastric junction was defined as the line where the squamous epithelium turns into a salmon-colored columnar epithelium. The patients were classified as normal esophagogastric junction (EGJ), short-segment BE, and long-segment BE. The Prague C & M classification system was used to document the extent of BE, measuring the circumferential extent (C) and the maximal extent (M) of columnar-lined epithelium visible above the EGJ. Displacement of the Z line 2 cm and above the proximal diaphragmatic hiatus was defined as HH. Reflux esophagitis detected during endoscopy was evaluated using the Los Angeles classification.

###  Pathological Evaluation

 Targeted biopsies were taken every 2 cm in four quadrants according to the Seattle protocol and processed using hematoxylin & eosin and Alcian blue staining. Two senior gastrointestinal pathologists evaluated the samples independently. Only cases showing intestinal metaplasia with goblet cells were classified as histologically confirmed BE. *H. pylori* infection was assessed by histopathological examination with special stains (e.g. Giemsa or Warthin-Starry) and confirmed by the pathologist. For patients using PPI, it is routinely recommended in our hospital to discontinue the medication at least 2 weeks prior to endoscopy to prevent false-negative results.

###  Statistical Analysis

 Statistical analysis was performed using SPSS 20. Chi-square or Fisher’s exact test were used for categorical variables. Variables were selected for multivariate logistic regression based on their clinical relevance and/or statistical significance in univariate analysis (*P*< 0.10), in order to balance interpretability and avoid model overfitting. A *P* value < 0.05 was considered statistically significant.

## Results

 The demographic and clinicopathological characteristics of the patients are shown in Table 1. Among the 840 patients with endoscopic suspicion of BE, 367 (44%) were female and 473 (56%) were male. The mean age was 51.0 ± 12.6 years (range 17–91). Short-segment BE was suspected in 818 patients (97.4%), and long-segment BE in 22 (2.6%).

**Table 1:** Demographic and Clinicopathological Characteristics of Patients

	**Female** **(n=367)**	**Male** **(n=473)**	**Total** **(N=840)**
Age. mean ± SD (range)	50.0 ± 12.5 (17‒90)	52.0 ± 12.8 (18‒91)	51.0 ± 12.6 (17‒91)
Smoking, n (%)	160 (43.6)	355 (75.1)	515 (61.3)
Alcohol, n (%)	48 (14.7)	147 (35.6)	195 (23.2)
Nausea/Vomiting, n (%)	266 (72.5)	282 (59.6)	548 (65.2)
Heartburn, n (%)	300 (81.7)	349 (73.8)	649 (77.3)
Epigastric pain, n (%)	157 (42.8)	166 (35.1)	517 (61.5)
PPI, n (%)	178 (48.5)	225 (47.6)	403 (48)
Hiatal hernia, n (%)	158 (43.1)	212 (44.8)	370 (44)
LES laxity, n (%)	179 (48.8)	251 (53.1)	430 (51.2)
Endoscopic esophagitis, n (%)	68 (18.5)	120 (25.4)	188 (22.4)
Gastritis, n (%)	306 (83.4)	407 (86)	713 (84.9)
Peptic ulcer, n (%)	23 (6.3)	41 (8.7)	64 (7.6)
Short-segment, n (%)	359 (97.8)	459 (97)	818 (97.4)
Long-segment, n (%)	8 (2.2)	14 (5)	22 (2.6)
Pathological gastritis, n (%)	156 (42.5)	235 (49.9)	391 (46.7)
Pathological esophagitis, n (%)	21 (5.7)	39 (8.2)	60 (7.1)
Adenocarcinoma, n (%)	0 (0)	4 (0.8)	4 (0.5)
Pathological BE, n (%)	191 (52)	232 (49)	423 (50.4)
Pathological *H. pylori*, *n* (%)	130 (35.4)	180 (38.1)	310 (36.9)

 Histologically confirmed BE was present in 423 patients (50.3%). BE confirmation was achieved in 414 (50.6%) of short-segment BE cases and in 9 (40.9%) of large-segment BE cases. HH was significantly more common in patients with confirmed BE (54.6% vs. 33.3%, *P* < 0.001), as was reflux esophagitis (27.7% vs. 17.0%, *P* < 0.001). No significant difference was found in rates of gastritis, peptic ulcer, or LES laxity (Table 2).

**Table 2:** Comparison of Patients with Histologically Confirmed Barret’s Esophagus and Non-Barret’s Esophagus Patients

	**Histologically confirmed Barret's esophagus (n=423)**	**Non-Barret's esophagus** **(n=17)**	***P *** **value**
Age. mean ± SD (range)	52.0 ± 12.4	50.0 ± 12.7	< 0.001
Female, n (%)	191 (45.1)	176 (42.2)	0.42
Male, n (%)	232 (54.8)	241 (57.7)	0.42
Smoking, n (%)	270 (63.8)	245 (58.7)	0.11
Alcohol, n (%)	110 (26)	85 (20.3)	0.07
Nausea/Vomiting, n (%)	280 (66.1)	270 (64.7)	0.85
Heartburn, n (%)	330 (78)	320 (76.7)	0.72
Epigastric pain, n (%)	260 (61.4)	257 (61.6)	0.93
PPI use, n (%)	205 (48.4)	198 (47.4)	0.77
Hiatal hernia, n (%)	231 (54.6)	139 (33.3)	**<0.001**
Endoscopic esophagitis, n (%)	117 (27.7)	71 (17.0)	**<0.001**
LES laxity, n (%)	210 (49.6)	220 (52.8)	**0.367**
Endoscopic gastritis, n (%)	356 (84.2)	357 (85.6)	**0.557**
Peptic ulcer, n (%)	34 (8)	30 (7.2)	**0.645**
Short segment BE	414 (97.9)	404 (96.9)	**0.369**
Long segment BE	9 (2.1)	13 (3.1)	**0.369**
Pathological gastritis	183 (43.3)	209 (50.1)	**0.046**
Pathological esophagitis	40 (9.5)	20 (4.8)	**0.009**
*H. pylori*	111 (26.2)	199 (47.7)	**<0.001**
Adenocarcinoma	3 (0.7)	1 (0.2)	**0.624**

BE, Barrett’s esophagus; LES, lower esophageal sphincter.

 Among patients with pathologically confirmed BE, *H. pylori* was detected in 26.2% compared to 47.7% in the non-BE group (*P* < 0.001). In patients with concurrent pathological gastritis, the frequency of *H. pylori* was higher in non-BE patients (66.3%) than in BE patients (46.4%, *P* < 0.001) (Table 3).

**Table 3:** Presence of *H. pylori* in Patients with Pathological Gastritis That Accompanies Patients with and without Histologically Proven Barret’s Esophagus

	**Pathological** **BE (n=183)**	**Pathological Negative** **BE (n=208)**	***P***
*H. pylori* positive	85 (46.4)	138 (66.3)	< 0.001
*H. pylori* negative	98 (53.6)	70 (33.7)	< 0.001

BE, Barrett’s esophagus.

 Multivariate regression analysis revealed that older age ( ≥ 50 years), male gender, presence of HH, and negative *H. pylori* status were independently associated with BE. Smoking, alcohol use, and presence of peptic ulcer were not statistically significant predictors (Table 4).

**Table 4:** Multivariate Logistic Regression Analysis of Factors Associated with Histologically Confirmed Barret’s Esophagus

**Variable**	**OR**	**95% CI**	***P *** **Value**
Age ≥ 50 years	1.67	1.34 – 2.09	< 0.001
Male sex	1.52	1.24 – 1.89	< 0.001
Hiatal hernia	2.35	1.86 – 2.96	< 0.001
*H. pylori* (present)	0.58	0.46 – 0.73	< 0.001
Smoking	1.12	0.89 – 1.41	0.33
Alcohol consumption	1.07	0.78 – 1.47	0.65
Lower esophageal sphincter laxity	0.93	0.73 – 1.18	0.54
Gastritis	0.96	0.74 – 1.24	0.75
Peptic ulcer	1.14	0.66 – 1.96	0.64

CI, confidence interval; OR, odds ratio.

## Discussion

 In our large-scale retrospective cohort, which included over 41,268 patients undergoing endoscopy for dyspeptic symptoms, the endoscopic prevalence of BE was found to be 2.03%, while histologically confirmed BE was present in 1.02% of all patients. These findings align with prior data from Middle Eastern and Turkish populations, which generally report lower BE prevalence compared to Western countries.^5,6^ The prevalence of BE varies widely in different populations, with reported rates ranging from 0.5% to 14%, depending on the study population, symptom profile, and diagnostic methodology.^7^ The observed discrepancy between endoscopic suspicion and histological confirmation of BE, particularly in short-segment cases, is consistent with previous studies, emphasizing the challenges in the endoscopic recognition of subtle mucosal changes and the variability in biopsy acquisition.^8^

 Age and male sex are well-established factors associated with BE. Consistent with existing literature, our study found that BE was more prevalent in male patients, though this gender difference did not reach statistical significance. The average age of BE diagnosis in our cohort was in the early 50s, slightly younger than Western cohorts where diagnosis often peaks in the sixth decade.^9^ This may reflect differences in referral patterns, healthcare access, or earlier onset of GERD symptoms in our population.

 A notable finding of our study was the strong association between HH and BE. HH is thought to promote GERD by disrupting the anti-reflux barrier, leading to prolonged acid exposure and mucosal injury. In a study by Cameron *et al.*, the incidence of HH in patients with BE was found to be 96% and 40% in the control group.^10^ In our cohort, the prevalence of HH was significantly higher in patients with histologically confirmed BE compared to non-BE patients. This demonstrates a strong association between HH and BE and suggests that its presence should raise clinical suspicion for BE during endoscopy.

 One of the most debated issues in recent years is the relationship between *H. pylori* infection and the development of BE. While *H. pylori* is a known cause of chronic gastritis and gastric intestinal metaplasia, its role in esophageal pathology is paradoxical. Several epidemiological studies have suggested a negative association between *H. pylori* and BE, proposing that *H. pylori*-induced corpus-predominant gastritis leads to hypochlorhydria, thereby reducing acid reflux and the risk of BE development.^11,12^

 Our data demonstrated an inverse relationship. We found that the prevalence of *H. pylori* was significantly lower in patients with BE compared to those without BE. Furthermore, among patients with histological gastritis, *H. pylori* positivity was significantly more common in those without BE than in those with BE, supporting the view that *H. pylori* infection may be linked with a reduced likelihood of BE, possibly through effects on gastric acid secretion. These findings are in line with meta-analyses suggesting an inverse association between *H. pylori* infection and the occurrence of BE and esophageal adenocarcinoma, particularly for CagA-positive strains, which are more likely to induce gastric atrophy and hypoacidity.^13,14^

 However, it is important to consider that the protective association might vary depending on *H. pylori* strain virulence, host genetic factors, and the topographic pattern of gastritis. Not all studies agree on the protective role of *H. pylori*, and some have raised concerns about potential confounding factors, such as PPI use and eradication therapy.^15^ Future prospective studies with serial pH monitoring and gastrin levels are needed to further clarify this interaction.

 Another interesting aspect of our study is the relatively high histopathological confirmation rate (50.3%) among patients with endoscopic suspicion of BE. Padda and Ramirez^8^ reported histopathological confirmation rates of 38% for short-segment and 75% for long-segment BE; our comparatively higher rate may be attributed to strict adherence to the Seattle biopsy protocol and evaluation by experienced gastrointestinal pathologists. Nevertheless, the remaining diagnostic gap underscores the need for standardized endoscopic techniques, such as high-definition endoscopy and virtual chromoendoscopy, to improve mucosal visualization and target biopsies more effectively.^3^

 Our study has limitations inherent to its retrospective design. Inter-observer variability among endoscopists and differences in biopsy techniques may have influenced diagnostic accuracy. Moreover, lack of data on symptom severity, duration of GERD, and PPI duration prior to endoscopy limits further stratification. Nonetheless, the large sample size and systematic pathological evaluation provide valuable insight into the prevalence and risk factors associated with BE in dyspeptic patients. In addition, potential selection bias (patients limited to a dyspeptic population, single-center data) and collider bias (as only patients with endoscopic suspicion of BE were included, which may influence associations with risk factors) should be considered when interpreting our findings.

## Conclusion

 This study confirms that BE, although less prevalent in our region compared to Western countries, remains an important clinical concern, particularly in male patients with GERD symptoms and HH. The inverse association between *H. pylori* infection and BE observed in our cohort supports the hypothesis that *H. pylori* may be linked with a lower prevalence of BE, meriting further investigation. Improving endoscopic detection through advanced imaging and strict biopsy protocols, along with awareness of positive association profiles, can enhance diagnostic accuracy and optimize surveillance strategies for BE.
